# Increased hypoxia-inducible factor 1α expression in lung cells of horses with recurrent airway obstruction

**DOI:** 10.1186/1746-6148-8-64

**Published:** 2012-05-23

**Authors:** Marie Toussaint, Laurence Fievez, Christophe J Desmet, Dimitri Pirottin, Frédéric Farnir, Fabrice Bureau, Pierre Lekeux

**Affiliations:** 1 Laboratory of Cellular and Molecular Physiology, GIGA-Research and Faculty of Veterinary Medicine, University of Liège, B34-Avenue de l’Hôpital, 1, 4000, Liège, Belgium; 2 Department of Animal productions, Faculty of Veterinary Medicine, University of Liège, B42-Boulevard de Colonster, 20, 4000, Liège, Belgium

**Keywords:** Hypoxia inductible transcription factor-1, Recurrent airway obstruction, Inflammation, Lung, Horse

## Abstract

**Background:**

Recurrent airway obstruction (RAO, also known as equine heaves) is an inflammatory condition caused by exposure of susceptible horses to organic dusts in hay. The immunological processes responsible for the development and the persistence of airway inflammation are still largely unknown. Hypoxia-inducible factor (Hif) is mainly known as a major regulator of energy homeostasis and cellular adaptation to hypoxia. More recently however, Hif also emerged as an essential regulator of innate immune responses. Here, we aimed at investigating the potential involvement of Hif1-α in myeloid cells in horse with recurrent airway obstruction.

**Results:**

In vitro, we observed that Hif is expressed in equine myeloid cells after hay dust stimulation and regulates genes such as tumor necrosis factor alpha (TNF-α), interleukin-8 (IL-8) and vascular endothelial growth factor A (VEGF-A). We further showed *in vivo* that airway challenge with hay dust upregulated Hif1-α mRNA expression in myeloid cells from the bronchoalveolar lavage fluid (BALF) of healthy and RAO-affected horses, with a more pronounced effect in cells from RAO-affected horses. Finally, Hif1-α mRNA expression in BALF cells from challenged horses correlated positively with lung dysfunction.

**Conclusion:**

Taken together, our results suggest an important role for Hif1-α in myeloid cells during hay dust-induced inflammation in horses with RAO. We therefore propose that future research aiming at functional inactivation of Hif1 in lung myeloid cells could open new therapeutic perspectives for RAO.

## Background

Recurrent airway obstruction (RAO) or heaves is a well-known respiratory disease in horses that shares any pathophysiological similarities with asthma in humans [1-3]. RAO is a severe, potentially debilitating, chronic inflammatory airway disease typically affecting middle-aged horses. Acute exacerbations are characterized by neutrophilic airway inflammation, coughing, periods of labored breathing at rest and exercise intolerance due to bronchospasm and mucus accumulation in the airways [4]. It is initiated following exposure to organic dusts, molds, and lipopolysaccharides (LPS) in hay [5]. Periods of acute exacerbation are interspersed by periods of remission, when horses are kept away from the causative environment [3]. The immunological processes responsible for the persistent airway inflammation are still largely unknown [6]. RAO is thought to result from an aberrant immune response orchestrated by antigen-specific T lymphocytes via the secretion of pro-inflammatory cytokines. Whether these T lymphocytes have a type 1 or type 2 phenotype and cytokine secretion profile is still a matter of debate [7-11]. Although little studied so far, innate immune mechanisms, which constitute a central interface between external stimuli and the adaptive immune system, may also play an important role in the pathophysiology of RAO.

Hif1 is an essential regulator of adaptation to low oxygen levels [12]. Hif1 is a heterodimer composed of an oxygen-regulated α-subunit and a constitutively expressed β-subunit. In general the abundance of α-subunits is primarily regulated by a family of prolyl hydroxylases (PHD). In normoxia, PHD is activated and directs the degradation of the α-subunit by the ubiquitin-proteasome pathway [13].

Under hypoxia, PHD activity decreases, which leads to the stabilization and translocation to the nucleus of the α-subunits which heterodimerize with the β-subunit. This dimer recognizes the hypoxia response elements (HREs) to induce target gene expression. Hif1 activity is primarily regulated by the abundance of the Hif1-α subunit [14]. Since Hif1 is highly involved in the adaptation of cellular metabolism to hypoxic condition, it was also proposed that Hif1 is an important promoter of inflammatory responses, which most often require innate immune cells to adapt to oxygen-deprived inflammatory environments [15,16]. Indeed, it was found that Hif1 regulates many pro-inflammatory genes such as tumor necrosis factor alpha (TNF-α), interleukin-8 (IL-8) and vascular endothelial growth factor A (VEGF-A) [17]. Hif1 was furthermore found to engage in cross-talk with another major pro-inflammatory pathway, the Nuclear Factor (NF)- κB pathway [18]. Although most studies focused on hypoxic conditions, Hif may promote inflammatory functions of myeloid cells also in normoxic conditions [19,20]. Indeed, Hif1-α may be indirectly activated in myeloid cells by pro-inflammatory stimuli such as LPS and the pro-inflammatory cytokine TNF-α [21,22]. After LPS or cytokine stimulation, NF-κB promotes Hif1-α gene transcription, promoting its accumulation in spite of post-translational degradation [18,23]. In myeloid cells, hypoxia through decreased Hif1-α degradation and inflammatory stimuli through increased Hif1-α transcription may synergistically potentiate the activation of Hif1.

We hypothesized that, because myeloid cells are implicated in RAO, and because Hif1 is a major regulator of the pro-inflammatory functions of myeloid cells, Hif1 could play a role in the pathophysiology of RAO. To test this hypothesis, complementary *in vitro* and *in vivo* approaches were undertaken.

Firstly we assessed *in vitro* expression of Hif1 mRNA and its target genes in horse myeloid cells in response to hay dust. Secondly, *in vivo*, we analyzed whether hay dust-induced lung inflammation in RAO-affected horses correlated with increased Hif1 expression in lung myeloid cells. Finally, we tested whether increased Hif1 expression in BALF myeloid cells correlated with clinical variables in RAO-affected horses.

## Results

### Hif1- is expressed basally and upregulated by hay dust in horse myeloid cells

**Figure 1:**
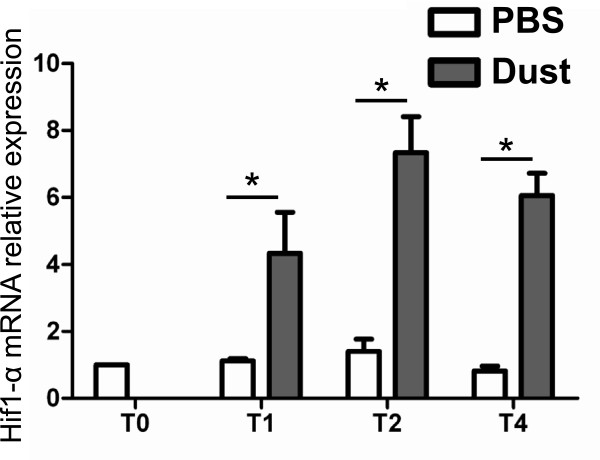
**Hif1-α mRNA relative expression (LSmean ± S.E.M) in equine monocytes 1, 2 and 4 hours after stimulation with hay dust (500 ng/mL).** The relative expression is normalized to the geometric mean of reference genes. This experiment is representative of three similar experiments. (*P < 0.05).

We first tested the presence of Hif1 in horse myeloid cells and assessed hay dust-induced Hif1 activation in these cells. Relative Hif1-α mRNA expression between excipient- and hay dust-treated monocytes was assessed 1, 2 and 4 hours after stimulation. The Hif1-α mRNA was expressed basally and this expression was significantly higher in stimulated than in unstimulated-cells 1 h after the beginning of the treatment, and maximal induction was achieved after a 2 h incubation period. At 4 h, Hif1-α expression was maintained at a high level (Figure 1).

### Hay dust induced pro-inflammatory genes associated with Hif1-α activity in equine monocytes

**Figure 2:**
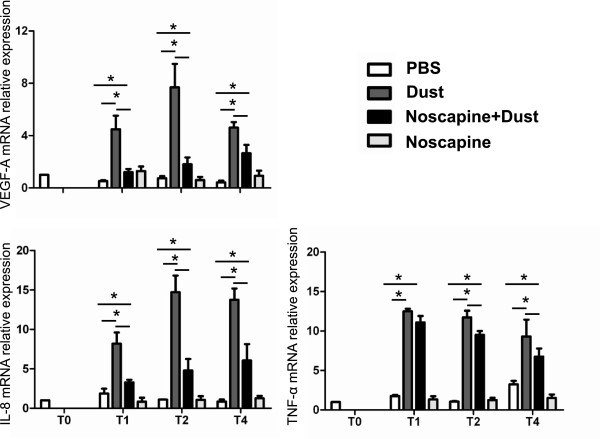
**VEGF-A , IL-8 and TNF-α mRNA relative expression (LSmean ± S.E.M) in equine monocytes 1, 2 and 4 hours after stimulation with a hay dust suspension.** Alternatively, cells were treated with noscapine (150 μM) one hour before stimulation with the hay dust suspension. The relative expression is normalized to the geometric mean of reference genes. This experiment is representative of three similar experiments. (*P < 0.05).

We next aimed at determining whether hay dust-induced Hif1-α mRNA upregulation may result in increased Hif1 activity in equine monocytes. Hay dust suspensions induced a significant increase in VEGF-A, IL-8 and TNF-α expression in equine monocytes as compared with excipient-treated monocytes (Figure 2). We observed strong positive correlation between Hif1-α induction and cytokine expression (Hif1-α/VEGF-A: r = 0.92; Hif1-α/IL-8: r = 0.86; Hif1-α/TNF-α: r = 0.81). Suggesting that the *in vitro* induction of cytokine expression was dependent on Hif1-α activity, noscapine, an inhibitor of the stabilization of Hif1-α, when added to the culture 1 h before challenge with hay dust, inhibited the induction of VEGF-A and IL-8. However, a lower effect of noscapine was observed for TNF-α. For IL-8, VEGF-A and TNF-α, some significant differences were still seen between excipient- and noscapine/hay dust-treated groups at all-time points tested. These data support that during hay dust stimulation, Hif1-α was partially required for VEGF-A and IL-8 expression in equine myeloid cells, whereas TNF-α induction was less dependent on Hif1. Noscapine alone had no effect on the expression of these three cytokines in equine monocytes (Figure 2).

### Horse clinical status assessment

**Figure 3:**
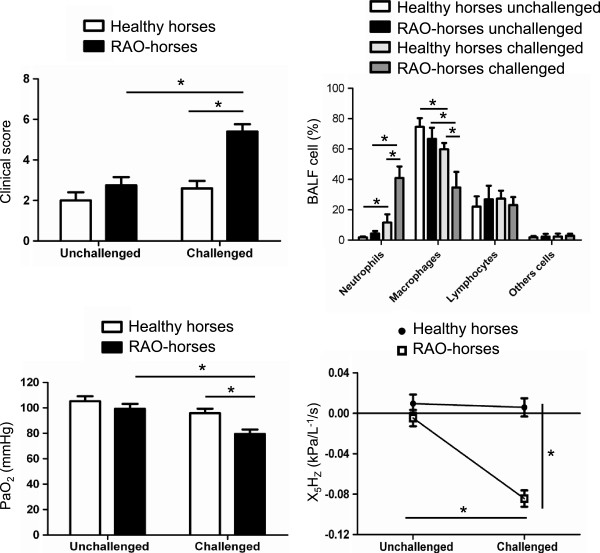
**Clinical parameters evaluated *in vivo *in unchallenged and challenged healthy and recurrent airway obstruction-affected horses**. Clinical score, percentage of cells in bronchoalveolar lavage fluid, arterial pressure of oxygen (PaO_2_) and lung function (X_5_H_Z_) were compared between the groups_._ This experiment is representative of one protocol performed with five heaves-affected horses and four healthy horses (*P < 0.05; RAO: recurrent airway obstruction).

Clinical scores were significantly different between both the healthy and RAO groups after dust exposure (Figure 3). In the BALF, neutrophil percentage was significantly increased in both healthy and RAO horses following hay dust challenge. However, challenged RAO-affected horses showed significantly higher neutrophil percentage than challenged healthy horses. After the challenge, the percentage of alveolar macrophage (AM) in the BALF was significantly decreased in RAO-affected and healthy horses but the decrease was significantly higher in RAO-affected horses than in healthy horses. No significant differences were observed between the percentages in lymphocytes and other cell types. After dust exposure, arterial blood oxygen partial pressure was significantly decreased in RAO-affected horses when compared to healthy horses. The most sensitive value of impulse oscillometry for RAO-affected horse evaluation, X_5_H_z_ was significantly different between RAO-affected horses and controls after challenge. Clinical values for each horse are shown in Table 1.

**Table 1:** Individual results of clinical evaluation

	**Horses**	**Neutrophils** **(%)**	**Lymphocytes** **(%)**	**Alveolar macrophages** **(%)**	**Epithelial cells (%)**	**Pa0_2_ (mmHg)**	**X_5_H_z_ (kPa/L-1/s)**	**Heave score/8**
Challenged	RAO 1	49	21	27	3	62	-0.112	7
	RAO 2	48	31	23	2	79	-0.091	6
	RAO 3	33	17	47	3	88	-0.045	4
	RAO 4	34	21	43	5	82	-0.067	5
	RAO 5	40.5	25	33	1.5	86	-0.107	5
	Healthy 1	10	24	62	4	105	0.021	2
	Healthy 2	12	31	58	1	98	-0.012	3
	Healthy 3	8	29	63	0	92	0.013	2
	Healthy 4	16	25	57	2	102	0.001	4
Unchallenged	RAO 1	5.5	33	60	1.5	86	-0.012	3
	RAO 2	6	16	77	1	92	-0.006	3
	RAO 3	4	29	65	2	98	0.007	2
	RAO 4	2	37	60	1	108	-0.012	2
	RAO 5	4.5	19	71	5.5	95	-0.001	3
	Healthy 1	2	23	73	2	98	0.028	2
	Healthy 2	1.5	19	78.5	1	104	-0.004	2
	Healthy 3	1	31	67	1	110	0.003	2
	Healthy 4	2.5	15	79.5	3	109	0.011	2

### Increased Hif1-α mRNA expression following hay dust challenge in BALF cells of healthy and RAO-affected horses

**Figure 4:**
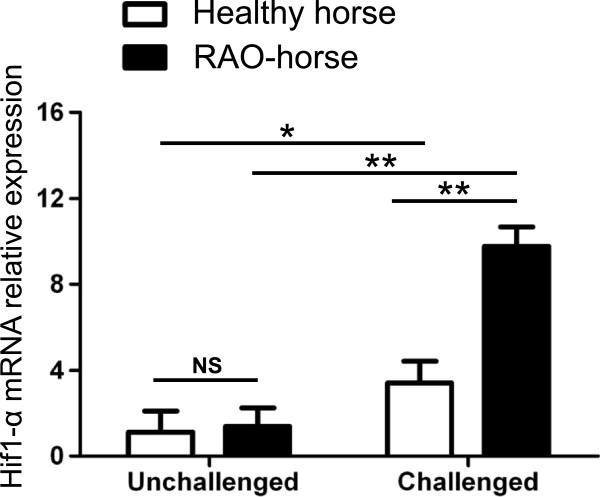
**The LSmean (±S.E.M.) Hif1-α mRNA relative expression in bronchoalveolar lavage cells of unchallenged and challenged healthy and recurrent airway obstruction-affected horses.** The relative expression is normalized to the geometric mean of reference genes. This experiment is representative of one protocol performed with five heaves-affected horses and four healthy horses (*P < 0.05;**P < 0.01; NS = no significant; RAO: recurrent airway obstruction).

To assess whether our in vitro observations may translate *in vivo*, we analyzed whether hay dust-induced lung inflammation in RAO-affected horses was accompanied by increased Hif1 activity in lung myeloid cells. We first compared the relative Hif1-α mRNA expression levels in BALF cells of unchallenged and hay dust-challenged healthy and RAO-affected horses. No statistically significant difference in Hif1-α expression was observed between unchallenged healthy and RAO-affected horses (Figure 4). Hif1-α mRNA levels significantly increased in both healthy and RAO horses following hay dust challenge. However, challenged RAO-affected horses showed significantly higher Hif1-α mRNA levels than challenged healthy horses.

### Hif1-α-regulated cytokines are overexpressed in the BALF cells of RAO-affected horses during crisis

**Figure 5:**
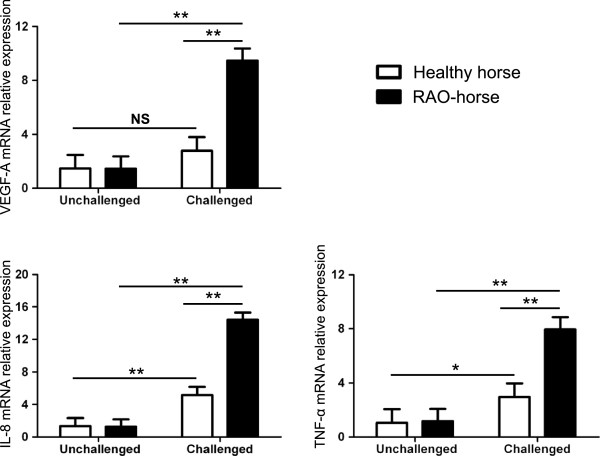
**The LSmean (±S.E.M) relative mRNA expression of the VEGF-A , IL-8 and TNF-α genes in bronchoalveolar lavage cells of unchallenged and challenged healthy and recurrent airway obstruction-affected horses.** The relative expression is normalized to the geometric mean of reference genes. This experiment is representative of one protocol performed with five heaves-affected horses and four healthy horses (*P < 0.05;**P < 0.01; NS = no significant; RAO: recurrent airway obstruction).

Next, the expression of Hif1 target genes was measured in BALF cells from hay dust-challenged or unchallenged healthy and RAO-affected horses (Figure 5). No statistically significant difference in VEGF-A, IL-8 and TNF-α expression was observed between the unchallenged groups. However, we observed that VEGF-A, IL-8 and TNF-α gene expression was upregulated following hay dust challenge. IL-8 and TNF-α mRNA expression were significantly increased in both healthy and RAO-affected horses upon challenge, whereas VEGF-A levels was upregulated in challenged RAO-affected but not healthy horses. We also observed positive correlations between Hif1-α mRNA expression levels and expression levels of its target genes (Hif1-α/VEGF-A: r = 0.86; Hif1-α/IL-8: r = 0.85; Hif1-α/TNF-α: r = 0.78).

### Correlation between Hif1-α expression in lung myeloid cells and lung dysfunction

We finally aimed at determining whether Hif1-α expression in lung myeloid cells correlates with lung dysfunction and inflammation. Table 2 shows that in challenged RAO-affected horses, Hif1-α mRNA expression levels were highly correlated to lung function (X_5_H_z_), arterial pressure of oxygen (PaO_2_)_,_ and the percentage of neutrophils in the BALF (% PMN). The correlations were positive for neutrophil percentages, and negative for X_5_H_z_ and PaO_2_. No correlations were observed in challenged healthy horses.

**Table 2:** Correlation between Hif1-α mRNA expression and clinical parameters

	**Status**	**Hif1-α mRNA/Neutrophils (%)**	**Hif1-α mRNA/PaO_2_ (mmHg)**	**Hif1-α mRNA/X_5_H_Z_ (kPa/L^-1^/s)**
Challenged	Healthy(n = 4)	r = −0.519	r = 0.231	r = −0.634
	RAO-affected(n = 5)	r = 0.863 (*)	r = −0.881 (*)	r = −0.844 (*)

Coefficient of correlations (r) between Hif1-α mRNA levels in bronchoalveolar lavage (BAL) cells and neutrophil percentages in BALF, reactance at 5 H_z_ (X_5_H_Z_), and arterial pressure of oxygen PaO_2_. These values were measured separately in challenged healthy and recurrent airway obstruction-affected horses. (*P < 0.05)

## Discussion

Hif1 is increasingly recognized as a master regulator of inflammatory responses. Recently, it has been demonstrated that Hif1 activity is increased in the lung of mouse models of asthma, as well as in BALF cells from asthmatic patients [24,25]. In the present work, we report similar findings in horses suffering from RAO, a disease that shares many similarities with human asthma. We demonstrated that the level of Hif1-α mRNA expression was significantly increased in BALF cells of hay dust-challenged horses and that this effect was more pronounced in RAO-affected than in healthy animals.

The increase in Hif1-α mRNA expression in BALF cells of challenged horses may be explained by the presence of LPS in hay dust. Indeed, it has been previously demonstrated that LPS induces Hif1-α mRNA expression through NF-κB activation in human monocytes and macrophages under normoxic conditions [26]. While administration of aerosolized LPS to RAO-affected horses induces airway neutrophilia, it is not sufficient to elicit an RAO crisis [5]. At the present time the contribution of LPS to disease severity remains unclear. Yet, emerging evidence suggests that a synergistic effect may exist between inhaled LPS and organic dust particulates. Hif1 activation could also be enhanced by other components present in the hay dust, such as Alternaria spores, β-Glucans, which also could activate NF- κB [27,28], even though this hypothesis will require further testing. From this first study, we suggest that LPS or other components present in hay dust may upregulate Hif1-α gene expression via NF-κB activation in both healthy and RAO-affected horses, with a more pronounced effect in RAO-affected horses. It is well known that NF-κB activation and hypoxia synergistically activate Hif1 [29]. Whereas NF-κB increases Hif1-α mRNA expression through fixation on a NF-κB site in the promoter of the Hif1-α gene, hypoxia post-translationally stabilizes the Hif1-α protein [29]. Accordingly, it has previously been shown that NF-κB activity is significantly increased in the lungs of RAO-affected horses during crisis when compared to unchallenged healthy horses [30]. Moreover, during RAO crises, inflammation induces a hypoxic environment in the lung tissues, which is a consequence of decreased perfusion, bronchial edema and increase in metabolic activity of recruited inflammatory cells. Furthermore, as illustrated by our clinical results, inflammation-induced local tissue hypoxia may be reinforced by the fact the horses in crisis usually become hypoxemic. Given that Hif1-α mRNA expression was significantly higher in challenged RAO-affected horses than in healthy subjects, it is possible that during the challenge, healthy horses, unlike RAO-affected horses, do not develop enough lung inflammation to induce hypoxia and thereby to increase Hif1 activity. A synergy between NF-κB activation and hypoxia in inflamed airways might thereby potentiate Hif1 activation in RAO. Further studies should be conducted to directly verify this hypothesis.

The observed increase in Hif1-α mRNA expression in BALF cells of hay dust-exposed horses did not necessarily imply that Hif1 transcriptional activity was increased. To estimate *in vivo* whether Hif1 was transcriptionally active, we could evaluated the expression of known Hif1 target genes. Hay dust challenge induced a significant increase in IL-8 and TNF-α mRNA expression in healthy and RAO-affected horses, and this increase was significantly more pronounced in RAO-affected horses. However, only challenged RAO-affected horses showed increased VEGF-A expression. The use of a Hif1-α inhibitor indicated that VEGF-A mRNA and IL-8 mRNA were highly regulated by Hif1 in hay dust stimulated-monocytes in vitro, in line with reports that Hif1 is able to regulate VEGF-A and IL-8 in human and murine macrophages [31]. However, the regulation of TNFα- mRNA expression in monocytes after hay dust stimulation was only weakly Hif1 dependent. It is likely that hay dust-induced TNF-α mRNA expression is mainly under the control of other transcription factor such as NF-κB. Following LPS stimulation, Hif1 can induce TNF-α transcription by direct fixation to the TNF-α gene promoter [32]. Yet NF-κB may also induce the transcriptional activation of TNF-α independently of Hif1 [33].

In an in vitro study, Laan and coll. [34] reported that IL-8 and TNFα- play a role in RAO. In equine AM stimulated with a hay dust solution, the expression of these two cytokines is significantly increased in RAO horses compared to healthy horses [34,35]. In rabbits, lung hypoxia was shown to promote IL-8 production from AM [36]. Moreover Franchini et al. [37] have suggested that AM are implicated in the initial inflammatory reaction, since they demonstrated that release of IL-8 by AM was required for neutrophil attraction. Taken together, these findings may suggest that in RAO-affected horses, the exacerbation of Hif1 activation in AM might be the initial response to airborne challenges, followed by an increase of IL-8 cytokine production and neutrophil invasion.

Currently, we have no information about the role played by VEGF-A in heaves. VEGF-A is a potent stimulator of vascular angiogenesis, permeability, and remodeling that also plays important roles in wound healing and tissue cytoprotection [38]. The regulation of vascular permeability is essential for inflammatory processes in airways, especially in the course of chronic lung diseases [39]. In humans beings and mice, VEGF-A is involved in the pathophysiology of bronchial asthma [39,40]. Given the similarities between RAO and asthma it may be speculated that VEGF-A could play an important role in RAO pathophysiology.

The expression of these three cytokines positively correlated *in vitro* and *in vivo* with that of Hif1-α. Even though our *in vitro* observations suggest that Hif1 activity in monocytes may contribute to expression of these cytokines *in vivo*, we do not rule out a possible involvement of Hif1 in other cell types.

Moreover even if monocytes are precursors of AM, they are not terminally differentiated cells. Therefore, some differences may exist in terms of regulation of Hif1 expression and activity between monocytes and AM. For instance, unlike AM, monocytes are unable to phagocyte and cytokine production and expression of surface molecules is sometimes different [41]. However, it is well known in others species that Hif1-α is expressed by AM [42] and that LPS can induce Hif1-α in macrophage [34,43]. It is thus highly likely that, like in monocytes, Hif1-α may be expressed by equine AM after hay dust challenge. Given the causative role proposed for AM in RAO, increased Hif1-α activity in AM following hay dust exposure might thus be responsible for crisis induction in RAO affected- horses.

It could also be worthwhile testing Hif1-α activity in equine neutrophils after hay dust stimulation. Indeed in mice, LPS stimulation can induce Hif1 activation in lung neutrophils [44]. After hay dust exposure, BALF neutrophil percentage increases only in RAO-affected horse and positively correlate with Hif1-α mRNA expression. Thus, it is possible that in RAO-affected horses, hay dust-induced neutrophil influx in the lung could also contribute to upregulated Hif1 activity.

Finally, we have also shown that in challenged RAO-affected horses the level of Hif1-α expression in BALF cells correlated with the degree of lung dysfunction. It will be worthwhile testing whether pharmacological inhibition of Hif1 in RAO-affected horses may impact on disease parameters. Supporting the relevance of this approach, it was shown that inhibition of Hif1 in mice using either pharmacological inhibition or conditional gene deletion significantly reduces airway inflammation in models of asthma [24,25].

## Conclusion

In summary, this study demonstrates that hay dust may activate Hif1 in horse myeloid cells. It further shows that lung myeloid cells display increased Hif1 activation upon hay dust challenge in RAO-affected horses compared to healthy horses, and that this activation correlates with disease severity. Our results thus suggest that it would be worthwhile testing the potential therapeutic benefit of pharmacological Hif1 inhibition in RAO.

## Methods

### Experimental animals

Five RAO horses with a history of recurrent episodes of respiratory distress when exposed to dusty stable environment (3 females and 2 geldings, median; ranges, 14.5; 9–22 years) and four healthy controls (1 female and 3 geldings, median; ranges, 17.5; 11–28 years) were investigated in the study. All horses affected by RAO showed recurrent clinical signs for several years and were therefore defined as chronically affected. Several months before the experiment began, horses were dewormed using ivermectin and vaccinated against tetanus and equine influenza. All experiments were conducted with approval of the Institutional Animal Care and Use Committee of the University of Liège.

### Experimental protocol *in vivo*

**Figure 6:**
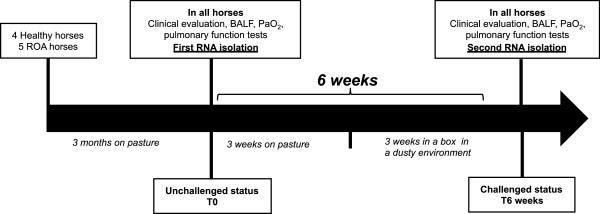
**Protocol *in vivo: *experimental design.**

Both groups of horses (RAO and control horses) were maintained on pasture 3 months before the beginning of the study to ensure the RAO horses were in clinical remission at the start of the experiment. All clinical and functional tests were performed on control and RAO horses in the steady-state (after pasture) and during the RAO crisis (3 weeks after the onset of the natural challenge with moldy hay) [45]. Six weeks separated the two testing periods (Figure 6). The clinical signs in horses were scored by a masked observer according to a previously reported system [46]. This protocol was approved by the Ethics Committee of the University of Liege.

### Pulmonary function tests

Pulmonary function was evaluated at rest with an equine impulse oscillometry system (IOS) Master Screen (VIASYS Healthcare GmbH, Höchberg, Germany), validated for the horse [47,48]. Only the reactance at 5 Hz was used because it is the most sensitive for measuring changes in mechanical ventilation in the lower respiratory system. Before each experiment, the system was calibrated and the tests were performed without any prior sedation.

### Arterial blood gas analysis

Arterial blood was anaerobically collected in heparinized syringes by arterial puncture of the carotid artery with a 20 G 0.9 x 40 mm needle (Terumo Europe, Leuven, Belgium). The blood was immediately analyzed for partial pressure of oxygen and carbon dioxide (PaO_2_ and PaCO_2_) with the use of an autocalibrated blood gas analyzer (OPTI CCA; Osmetech, Roswell, Georgia, USA). The results were corrected for the patient’s body temperature.

### Bronchoalveolar lavage fluid cell isolation

The horses were sedated with IV romifidine, (0.01 mg/kg; Sedivet; Boehringer Ingelheim, Ingelheim, Germany), and butorphanol (0.02 mg/kg; Torbugesic; Fort Dodge, Wyeth, Madison, New Jersey, USA). Bronchoalveolar lavage was performed by instillation of six boluses of 60 mL of sterile saline with EDTA (0.6 mM) at 37°C into the working channel of a fiberoptic endoscope (250 cm x 9 mm, Pentax, Breda, The Netherlands) wedged into a bronchus. The samples recovered from the six syringes were pooled and immediately placed on ice. An aliquot was collected for cytological analysis and differential cell counts.

Bronchoalveolar lavage fluid cells were collected by centrifugation for 7 min at 300 g. The pellet was filtered and washed twice in phosphate buffered saline (PBS). Cells were resuspended in lysis buffer RA1 (NucleoSpin® RNAII; Macherey-Nagel, Düren, Germany) according to the manufacturer’s instructions and stored at −80°C until RNA isolation.

### Production of hay dust suspension

Hay dust suspensions were prepared as previously described by Pirie et al. [49]. Briefly, visibly moldy patches of hay were manually agitated and the dust collected. Fine dust (1 g) was suspended in 1 mL isotonic saline (0.9% NaCl) solution and assayed for LPS content. The quantity of LPS present in the hay dust suspension (48234 EU/mL) was measured by ELISA (ENdoLISA®; Hyglos, Bernried am Starnberger See, Germany). The hay dust suspension was diluted in culture medium to a concentration of 1 μg/mL (LPS content: 48.234 EU/mL).

### Isolation of peripheral blood monocytes

Because they required large numbers of cells, these experiments were conducted on equine monocytes as a model of equine myeloid cells. Blood was collected from three healthy adult horses into 2 mM EDTA. The blood was diluted twice with PBS and layered on Histopaque-1077 (Sigma Diagnostics, St Louis, MO). After centrifugation at 400 g for 25 min at 4°C, the mononuclear cells were aspirated and washed twice with ice-cold PBS-EDTA. The cell pellet was suspended in RPMI-1640 with 10% inactivated fetal bovine serum (FBS), penicillin (50 U/mL), and streptomycin (50 μg/mL) and after was incubated at 37°C in 5% CO2 for two hours to permit adherence of monocytes. Nonadherent cells were removed by three washes with PBS. The adherent cell population consisted of >85% monocytes. The monocytes were re-suspended in RPMI-1640 (1×10^6^cells/mL) and seeded in a 6-well culture plate. Each well received 1 of the following 4 treatments: PBS, hay dust suspension (1 μg/mL), Noscapine hydrochloride (150 μM; Sigma-Aldrich, Saint Louis, MO, USA) or Noscapine hydrochloride (150 μM) combined with a hay dust suspension (1 μg/mL). In order to model a stimulation as close as possible to the *in vivo* situation, we chose to stimulate monocytes with hay dust suspensions.

**Figure 7:**
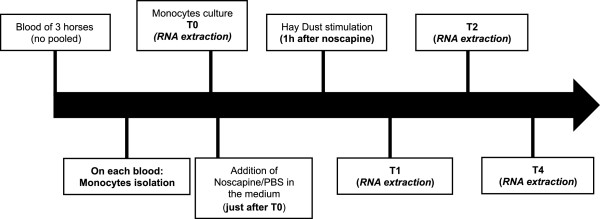
Protocol in vitro: experimental design.

Noscapine, an inhibitor of Hif1 activity, was added 1 hour before the hay dust suspensions (Figure 7). [50,51] One, two and four hours after treatments, the monocytes were collected, washed with PBS and lysed in buffer RA1 (NucleoSpin® RNAII; Macherey-Nagel, Düren, Germany) according to the manufacturer’s instructions and stored at −80°C until RNA isolation.

### RNA isolation from blood monocytes and BALF cells and real-time RT-PCR

Total RNA from blood monocytes and BALF cells was extracted using NucleoSpin®RNAII (Macherey-Nagel, Düren, Germany) in presence of Dnase.

Total RNA quantity was measured using a Nanodrop® ND-1000 spectrophotometer (Isogen Life Science B.V., Netherlands). One microgram of RNA was used for each reverse transcription using the First Strand cDNA Synthesis kit (Roche, Basel, Switzerland).

Quantitative PCR reactions were performed with IQ Sybr Green Supermix (Bio-Rad Laboratores, Inc., Marne-la-Coquette, France). The reaction master mix was prepared as recommended by the manufacturer.

To determine whether hay dust-induced Hif1α mRNA upregulation results in increased Hif1 activity in equine monocytes. We evaluated Hif1 activation by studying the level of expression of the Hif1 target genes VEGF-A, IL-8 and TNF-α. We stimulated or not equine monocytes with hay dust and relative expression of VEGF-A, IL-8 and TNF-α was assessed by RT-qPCR. Primers for the targets: Hif1-α, IL-8, VEGF-Alpha, TNF-alpha and reference genes: ACTB (β-actin), GAPDH (glycéraldhéyde-3 phosphate dehydrogenase), HPRT (hypoxanthine phosphoribosyltransferase), RPL32 (ribosomal protein L32) and SDHA (succinate dehydrogenase complex subunit A) were designed with Primer3Plus software and purchased from Eurogentec (Liège, Belgique). For each investigated gene, GenBank accession numbers, primer sequences and product size are given in Table 3. Due to lack of sequence in NCBI database, horse RPL32 sequence was retrieved by probing UCSC horse genome sequence with human RPL32 mRNA sequence. Primers were then designed in conserved regions by using Primer3Plus software.

**Table 3:** Primer sequences used for RT-qPCR validation procedure

**Genes**	**Equine accession number**	**Primers sequence**		**Product size (bp)**
		5′--------------------------------- > 3′		
Targets				
Hif1-α	LOC100061166	Forward	ctcaaatgcaagaacctgctc	108
		Reverse	ttccataccatcttttgtcactg	
VEGF-α	NM_001081821	Forward	tggcagaaggagagcataaaa	123
		Reverse	actcgatctcatcggggtact	
IL-8	NM_001083951	Forward	aatgagagcgattgagagtgg	127
		Reverse	caaaaacgcctgcacaataat	
TNF-α	EU438779	Forward	agcctcttctccttcctcctt	123
		Reverse	cagagggttgattgactggaa	
Housekeeping gene				
ACTB	NM_001081838.1	Forward	ggacctgacggactacctc	81
		Reverse	cacgcacgatttccctctc	
GAPDH	NM_001163856	Forward	atctgacctgccgcctggag	70
		Reverse	cgatgcctgcttcaccaccttc	
HPRT	XM_001490189.2	Forward	aattatggacaggactgaacgg	121
		Reverse	ataatccagcaggtcagcaaag	
RPL32	None	Forward	gggagcaataagaaaacgaagc	113
		Reverse	cttggaggagacattgtgagc	
SDHA	XM_001490889.3	Forward	gaggaatggtctggaatactg	91
		Reverse	gcctctgctccataaatcg	

The cDNA were amplified and quantified in the ICycler iQ real-time PCR detection system (Bio-Rad Laboratories N.V., Nazareth Eke, Belgium). At the end of each qPCR, a melt curve was generated to confirm that a single sequence was amplified for each primer pair. Agarose gel electrophoresis and the melting curve were used to assess the specificity of gene amplification. To confirm reproducibility, all samples were analyzed in duplicate on the plate. The entire experiments were performed in triplicate on independent samples. For each qPCR data analysis, reference genes were analyzed in parallel.

### Data analysis

Real-time qPCR results were analyzed using the QBASE + software (Biogazelle, Ghent, Belgium), including the geNorm tool to assess reference genes stability (Table 4). Internal control genes were ranked according to their gene-stability measure M. M is computed by determining, for every control gene, the average pairwise variation with all other control genes as the standard deviation of the logarithmically transformed expression ratios [52]. Genes with the lowest M values have the most stable expression. This method constitutes an improvement over the classical delta-delta-Ct method, since it takes into account multiple reference genes, resulting in improved data normalization. The values obtained from the in vitro and *in vivo* study were normalized with the two most stable reference genes RPL32 and HPRT.

**Table 4:** Reference genes stability according to geNorm

**Gene symbol**	**Stability value** **in vitro (M)**	**Stability value** ***in vivo* (M)**
HPRT	0.489	0.594
SDHA	0.784	0.711
RPL32	0.565	0.512
GAPDH	0.625	0.888
ACTB	0.883	0.904

Genes with the lowest M values have the most stable expression.

### Statistical analysis

All data are presented as least square means (LSmeans) ± associated S.E.M. (standard error of the mean) for quantitative PCR results. The residual distribution was normally distributed.

During the in vitro study, all four genes were analyzed using the same mixed model (SAS, Cary, NC). The models included the “time” factor (with levels T0, T1, T2 and T4), the “treatment” factor (PBS, dust, noscapine, dust + noscapine) and the interaction between these two factors. There was no effect of “time” in the in vitro kinetic. Correlations between successive measurements made on the same individual were taken into account using an auto-regressive residuals structure. The in vitro experiments were repeated at least three times. P < 0.05 was considered significant.

The clinical values, were analyzed using the PROC GLM procedure of the SAS package (SAS Institute, Cary, NC) and a model including “disease status” (healthy vs RAO), “treatment” (no challenged vs challenged), and their interactions as fixed effects. Separate estimates of the least square means of the states, the treatments and the interaction between the disease status and treatments effects have been obtained for each clinical parameter. P <0.05 was considered significant.

During the *in vivo* experiment, relative expression values, as obtained from qBasePlus software, were analyzed using the PROC GLM procedure of the SAS package (SAS Institute, Cary, NC) and a model including “disease status” (healthy vs RAO), “treatment” (no challenged vs challenged), "gene" (IL-8, Hif1, TNF-α, VEGF-A) and their interactions as fixed effects. Separate estimates of the least square means of the states, the treatments and the interaction between the disease status and treatments effects have been obtained for each gene". P <0.05 was considered significant.

To study the correlation between the genes expression and the physiological parameters (the percentage of neutrophils, X_5_H_z_ and PaO_2_) standard least-square linear regressions were carried out. Coefficients of correlation of pearson (r) were presented as measures of linear association for regression relationships. Significant differences of the slopes from zero were determined using a two-tailed Student's *t* test. P <0.05 was considered significant.

## Abbreviations

ACTB: β-actin; ATP: Adenosine triphosphate; B2M: β-2-microglobulin; BALF: Bronchoalveolar lavage fluid; GAPDH: Glycéraldhéyde-3 phosphate dehydrogenase; Hif: Hypoxia-inducible factor; HPRT: Hypoxanthine phosphoribosyltransferase; HRE: Hypoxia response element; IL-1β: Interleukin 1-β; IL-8: Interleukin 8; IOS: Impulse oscillometry system; LPS: Lipopolysaccharides; AM: Alveolar macrophage; NF- B: Nuclear factor B; Pa02: Arterial blood pressure oxygen; PBS: Phosphate buffered saline; PHD: Prolyl hydroxylases; PMN: Polymorphonuclear neutrophils; RAO: Recurrent airway obstruction; RPL32: Ribosomal protein L32; SDHA: Succinate dehydrogenase complex subunit; TNF-α: Tumor necrosis factor alpha; X5HZ: Lung resistance.

## Competing interest

This manuscript, in whole or in part, has not been published elsewhere, and has been read and approved by all authors. Sources of extra institutional funding or support have been acknowledged. There are no financial or personal conflicts of interest. The authors represent and warrant that our part of the work as submitted will in no way violate any copyright, or any other right. The authors declare that they have no competing interests.

## Authors' contributions

MT performed most of the experiments. LF helped to isolate the equine monocytes. DP participated in the qBase and statistical analysis. FB, PL, participated in the design of the study. FF performed the statistical analysis. CD helped to draft the manuscript. All authors read and approved the final manuscript.
